# Femtosecond Laser Introduced Cantilever Beam on Optical Fiber for Vibration Sensing

**DOI:** 10.3390/s24237479

**Published:** 2024-11-23

**Authors:** Jin Qiu, Zijie Wang, Zhihong Ke, Tianlong Tao, Shuhui Liu, Quanrong Deng, Wei Huang, Weijun Tong

**Affiliations:** 1 Hubei Key Laboratory of Optical Information and Pattern Recognition, Wuhan Institute of Technology, Wuhan 430205, China; qiujin@wit.edu.cn (J.Q.); 22210010067@stu.wit.edu.cn (Z.W.); 22210010046@stu.wit.edu.cn (Z.K.); 22210010061@stu.wit.edu.cn (T.T.); quanrongdeng@wit.edu.cn (Q.D.); huangw@wit.edu.cn (W.H.); twj@wit.edu.cn (W.T.); 2 Wuhan Fibersight Optoelectronic Science and Technology Corporation Ltd., Wuhan 430200, China

**Keywords:** fiber vibration sensor, Fabry-Perot interferometer, femtosecond laser micromachining

## Abstract

An all-fiber vibration sensor based on the Fabry-Perot interferometer (FPI) is proposed and experimentally evaluated in this study. The sensor is fabricated by introducing a Fabry-Perot cavity to the single-mode fiber using femtosecond laser ablation. The cavity and the tail act together as a cantilever beam, which can be used as a vibration receiver. When mechanical vibrations are applied, the cavity length of the Fabry-Perot interferometer changes accordingly, altering the interference fringes. Due to the low moment of inertia of the fiber optic cantilever beam, the sensor can achieve broadband frequency responses and high vibration sensitivity without an external vibration receiver structure. The frequency range of sensor detection is 70 Hz–110 kHz, and the sensitivity of the sensor is 60 mV/V. The sensor’s signal-to-noise ratio (SNR) can reach 56 dB. The influence of the sensor parameters (cavity depth and fiber tail length) on the sensing performance are also investigated in this study. The sensor has the advantages of compact structure, high sensitivity, and wideband frequency response, which could be a promising candidate for vibration sensing.

## 1. Introduction

Vibration sensors play an essential role in many fields, including aerospace engineering, structural health monitoring, civil engineering, seismic detection, equipment maintenance, and fault detection [1,2,3,4,5]. Piezoelectric vibration sensors are one of the most commonly used sensors [6], which, however, perform poorly under extreme conditions like electromagnetic interference. On the other hand, optical fiber vibration sensors offer features such as resistance to electromagnetic interference, small size, light weight, and high sensitivity. These unique advantages have led to their increasingly widespread use in the field of vibration measurements [7,8,9,10].

Despite these benefits, the small size and light weight of optical fibers often necessitate complex external structures to enhance vibration signal capture and improve sensor sensitivity. This complexity increases both the volume and the intricacy of the sensor. For example, sensors based on fiber Bragg grating often require additional mechanical structures, such as cantilever beams and mass blocks, to enhance sensitivity [11,12,13,14,15]. Similarly, a fiber optic vibration sensor based on the Mach-Zehnder interferometer (MZI) at the dispersion turning point incorporates a rectangular cantilever structure [16]. Zhao et al. developed an optical fiber vibration sensor using tapered hollow core fiber (HCF) within an MZI, which also relies on an additional cantilever beam [17]. These external structured sensors tend to be large, complex, and costly. Furthermore, their measurement frequency ranges are typically narrow, as seen in a study by Jia et al., where the sensor’s x/y axis operation frequency range was 60–150 Hz and 30–150 Hz, respectively [13].

There are also all-fiber vibration sensors that, while avoiding complex external structures, utilize various types of optical fibers, resulting in more complicated fabrication processes and higher costs [18,19,20,21]. On the other hand, internal structures have been fabricated inside optical fibers to measure refractive index [22,23], curvature, and temperature [24,25], which would also be promising to be used as a vibration sensor.

In this study, we introduce an all-fiber vibration sensor based on a fiber optic Fabry-Perot interferometer (FPI). Femtosecond laser is used to fabricate an in-fiber cavity on a single-mode fiber, forming the FPI. The in-fiber cavity reduces the moment of inertia of the fiber tail, allowing it to act as a fiber optic cantilever for receiving external vibration signals. Vibration causes significant changes in the cavity length of the FPI, leading to variations in the sensor’s signal light intensity. We conducted vibration sensing experiments on the proposed sensor, and the experimental results indicate that the sensor exhibits high vibration sensitivity and a broadband frequency response. The sensor has a measurement frequency range from 70 Hz to 110 kHz, a sensitivity of 60 mV/V, and an SNR of 56 dB. We also investigated the impact of the sensor parameter (cavity depth and the fiber tail length) on sensor performance. Our design leverages an all-fiber groove structure, providing the advantages of small size, simple structure, low cost, high sensitivity, and a broad measurement range.

## 2. Sensor Design and Manufacture

**Figure 1:**
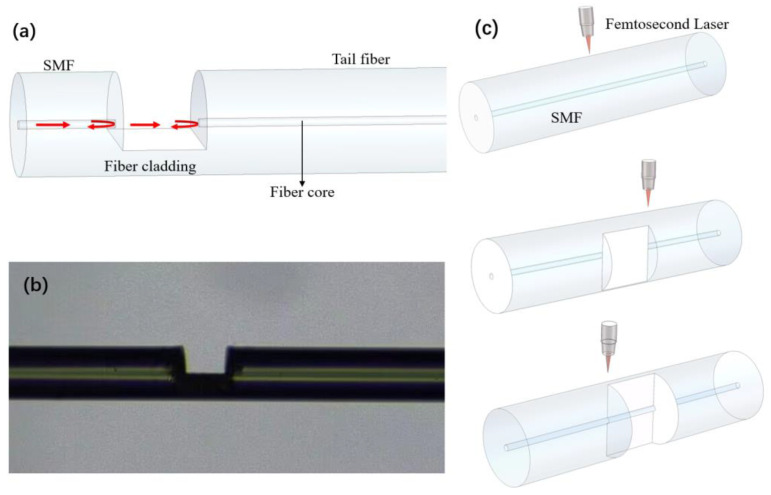
(**a**) The structure of the sensor. (**b**) Microscope image of the sensing structure. (**c**) The manufacturing process of the sensor.

The structure of our proposed sensor is shown in Figure 1a. The sensor is fabricated by removing part of the fiber material of a single-mode fiber using femtosecond laser ablation to form an in-fiber FP cavity. Fiber tails with a length of several millimeters are left on the far end of the FPI, which act as cantilever beams of the sensor. The incident light is reflected by the two mirrors of the FPI, as shown in Figure 1a, which leads to interference in the reflection spectrum. The interference spectrum is determined by the cavity length of the FPI, which can be affected by vibration. Figure 1b shows the microscope image of a fabricated sensor. The fabrication process of the sensor is shown in Figure 1c. 

**Figure 2:**
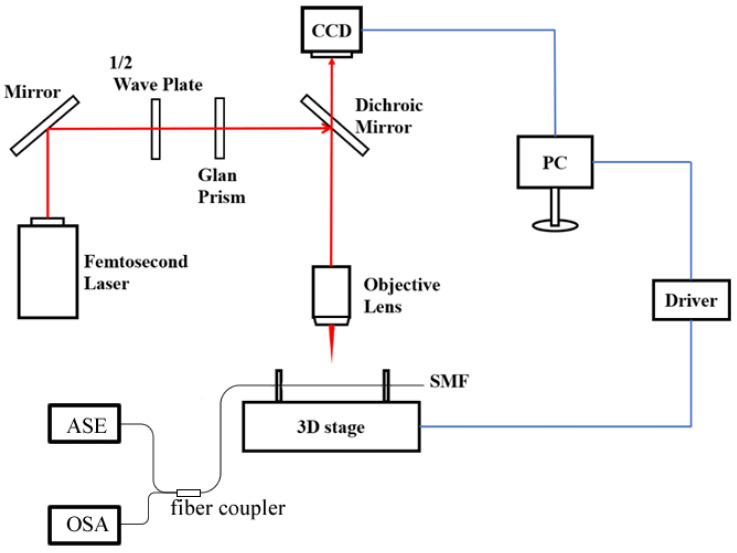
Experimental set up for the fabrication of the in-fiber cavity.

Figure 2 illustrates the experimental set up for the fabrication of the in-fiber cavity. A Ti:sapphire Femtosecond laser (Spectra Physics, Irvine, CA, USA, Solstice, λ = 800 nm, 100 fs, 1 kHz) is used to fabricate the sensor. The power of the laser is tuned by a halfwave plate followed by a Glan lens. Laser pulses with a pulse energy of 20 μJ is focused on the fiber through a 10×, NA = 0.25 objective lens (Nikon, Tokyo, Japan) with a working distance of 7 mm, as illustrated in Figure 2. A CCD camera is used to monitor the fabrication process in real time. A standard single-mode optical fiber (YOFC, Wuhan, China) is fixed on a high precision translation stage with a resolution of 50 nm. The translation stage is controlled by a computer to move along the fiber length at a speed of 20 µm/s, while part of the fiber material is ablated by the focused laser. After each ablation cycle, the focusing point is moved closer to the fiber core with a distance of 1 µm, before the next cycle. An in-fiber cavity of the desired length and depth can be created in the single-mode fiber after several cycles of laser ablation. Then, the femtosecond laser is used to clean the debris on the mirror, which could increase the reflectivity on interfaces, and further lead to higher fringe visibility in the reflection spectrum. Finally, the far end of the fiber is cut to leave a fiber tail with certain length to complete the sensor sample. Samples with different cavity depths (75, 70, and 65 µm), and different fiber end lengths (15, 10, and 5 mm) are fabricated experimentally.

**Figure 3:**
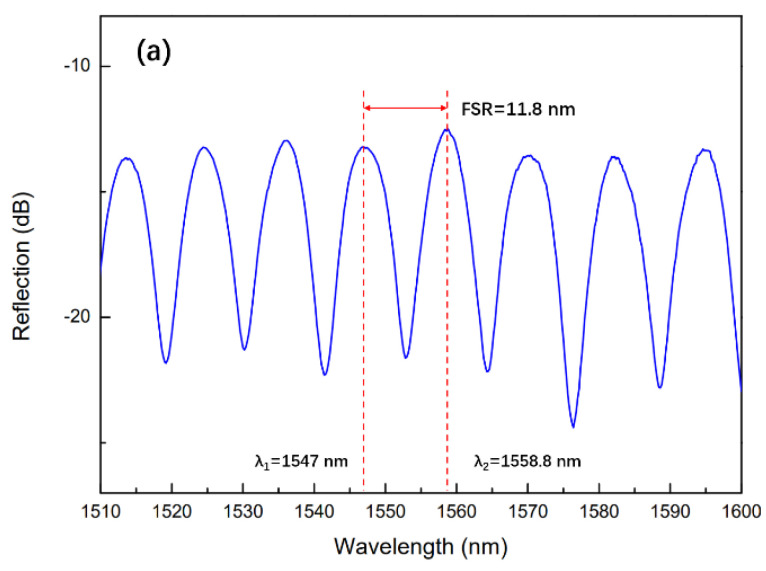

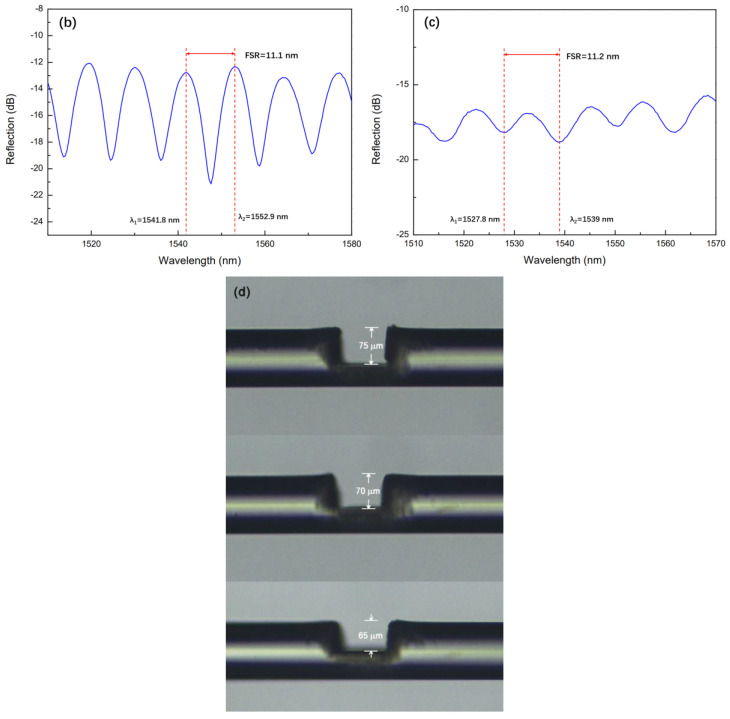
Reflectance spectra of sensors with cavity depths of 75 µm (**a**), 70 µm (**b**), and 65 µm (**c**). (**d**) Microscope images of the sensors with different cavity depths.

The reflectance spectrum of the sensor is measured by an amplified spontaneous emission (ASE) light source with wavelength ranging from 1540 nm to 1600 nm and an optical spectrum analyzer (OSA, YOKOGAWA, AQ6370C, Tokyo, Japan) through an optical fiber coupler. Figure 3a shows the reflection spectrum of the sensor with a cavity depth of 75 µm. It can be seen that the reflection spectrum has a high fringe contrast. Similarly, we also tested the reflectance spectra of sensors with cavity depths of 70 and 65 µm, and the test results are shown in Figure 3b,c. As can be seen from the figure, the contrast of the reflection spectrum of the sensor with a cavity depth of 70 µm is slightly lower compared to the sensor with a cavity depth of 75 µm. That is because the sensor with depth of 75 µm has higher reflectivities at both reflection mirrors. As for the sensor with a cavity depth of 65 µm, the fringe contrast is the lowest among the three samples, since only part of the core is removed, resulting in only a portion of the incident light taking part in the FP interference. The microscope images of these samples are shown in Figure 3d.

Because of the low reflectance of the two reflecting surfaces, higher-order reflections are negligible. The FPI structure of the sensor can be approximated as a two-beam interferometer. The spectral intensity of FPI can be expressed as,

(1) $$I=I_{1}+I_{2}+2\sqrt{I_{1}I_{2}\cos\varphi }$$

where I_1_ and I_2_ are the light intensity of the reflected light generated after the light passes through the reflecting surface, respectively. φ is the phase shift between two reflected beams. The calculation of φ is given as follows:

(2) $$\varphi =\frac{4\pi n_{air}L}{\lambda }$$

where n_air_ is the refractive index of air at room temperature. The FSR of the interference fringe, which is given in Equation (3), is determined by the wavelength λ, the effective refractive index n_eff_ of the FP cavity, and the cavity length L of the FP cavity.

(3) $$FSR=\frac{\lambda^{2}}{2n_{eff}L}$$

The FSR of the sensor read out in Figure 3a is 11.8 nm. Since the FP cavity of the sensor is an air cavity and the refractive index of air at room temperature is 1.00029, the cavity length L of 102.4 µm can be obtained by using Equation (1). This is approximately consistent with the cavity length (~100 µm) of the sensor we made.

## 3. Experimental Results and Discussion

### 3.1. Experimental Platform Construction

**Figure 4:**
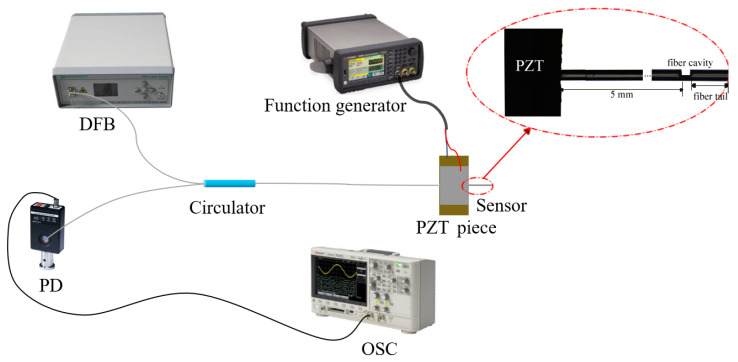
Vibration sensing experiment platform. Inset is a microscope image of the fiber sensor fixed at the PZT. (Note that the distance from the fiber cavity to the edge of the PZT is 5 mm, and we cannot visualize the whole structure in such scale with the microscope. The inset comprises two microscope images separated by an ellipsis to illustrate the whole structure).

We set up an experimental platform for vibration measurement to verify the feasibility of this sensor for vibration sensing. As shown in Figure 4, we fixed the cavity vibration sensor on a piezoelectric ceramic (PZT) with glue. Considering that the orientation of the cavity has direct influence on the vibration sensing, the cavity is oriented to the oscillation direction of the PZT to achieve better sensitivity. The distance from the fiber cavity to the edge of the PZT is 5 mm, as shown in the inset of Figure 4. The PZT is connected to a function generator (KEYSIGHT, Santa Rosa, CA, USA), which is used to control the vibration signal. The cavity is fixed 5 mm away from the edge of the PZT, as shown in Figure 4. The sensor is connected to the optical circulator; the other two ends of the optical circulator are connected to a DFB Laser and a photodetector (PD). The PD converts the optical signal into voltage signal, which is collected by an oscilloscope (KEYSIGHT) for observing the vibration signal.

### 3.2. Vibration Sensing Experiment

**Figure 5:**
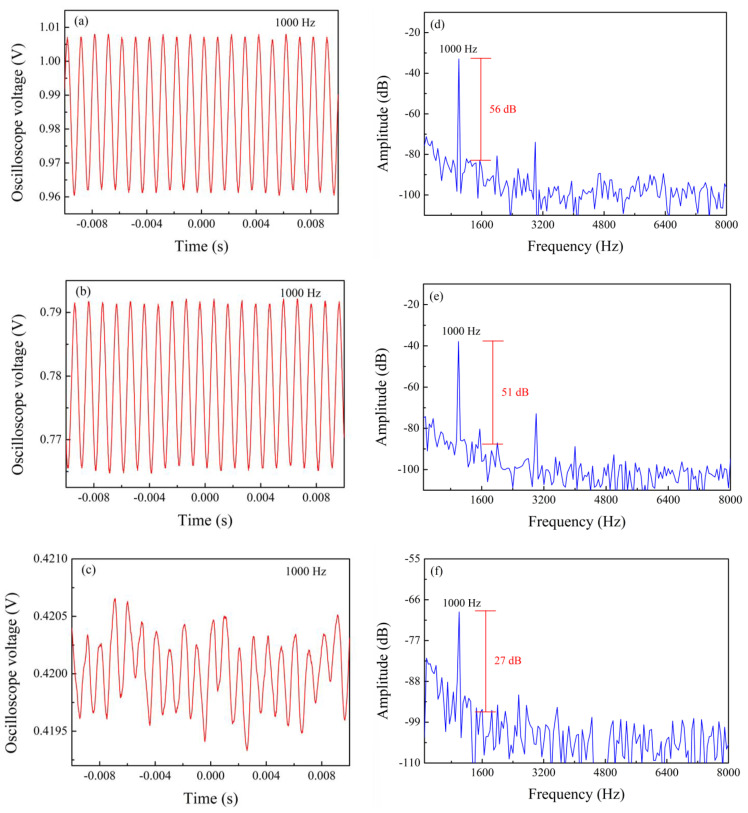
Time domain diagram (**a**–**c**) and frequency domain diagram (**d**–**f**) of sensors with cavity depths of 75, 70, 65 µm for a frequency of 1000 Hz and a voltage amplitude of 10 V.

**Figure 6:**
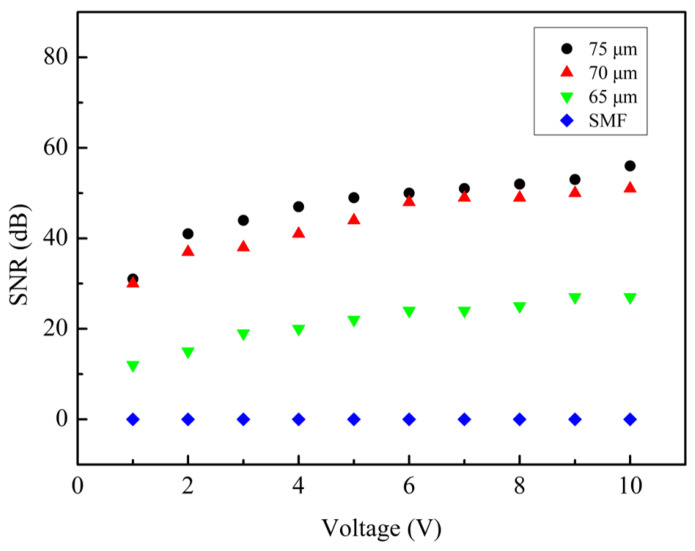
The SNR of sensors with cavity depths of 75, 70, and 65 µm, when the voltage is adjusted from 1 V to 10 V (the frequency is 1000 Hz).

We first studied the influence of cavity depths on the sensor performance. Vibration sensing experiments were performed on sensors with groove depths of 75, 70, and 65 µm, respectively. The tail length of these sensors is 15 mm. During the experiment, the sine wave frequency of the function generator was fixed at 1000 Hz, the voltage amplitude of the function generator was varied from 1 V to 10 V, and the data was recorded every 1 V. Figure 5 shows the time domain and frequency domain diagrams of sensors with cavity depths of 75, 70, 65 µm when the function generator voltage amplitude is 10 V. Their SNRs of the three sensors were 56, 51, and 27 dB, respectively. After a fast Fourier transform (FFT) algorithm was applied to these results, we obtained the signal-to-noise ratio changes of sensors with cavity depths of 75, 70, and 65 µm under different applied voltages, which are plotted in Figure 6. In order to exclude the influence of other factors, we also carried out the above experiments on the unprocessed single-mode fiber; the SNR of the obtained single-mode fiber was 0 (Figure 6). As can be seen from the figure, the sensor with a cavity depth of 65 µm has the lowest signal-to-noise ratio, which is lower than 40 dB. That is because the sensor with a cavity depth of 65 µm only grinds away part of the core during processing, and the reflectivity at the interfaces are weak, leading to quite low fringe visibility, as can be seen from Figure 3a. The reflected signals will undergo very small intensity variation during the vibration process, and the SNR will be low. On the other hand, when the depth of the cavity is larger, the moment of inertia of the sensor is greater. Thus, the sensor is easier to deform under vibration, and the FP cavity stretches in a larger scale. Accordingly, the sensor with a cavity depth of 75 µm has the highest fringe visibility according to Figure 3c, and thus the signal-to-noise ratio is also the highest, as can be seen from Figure 6. We therefore chose a cavity depth of 75 µm structure to continue the following vibration test.

**Figure 7:**
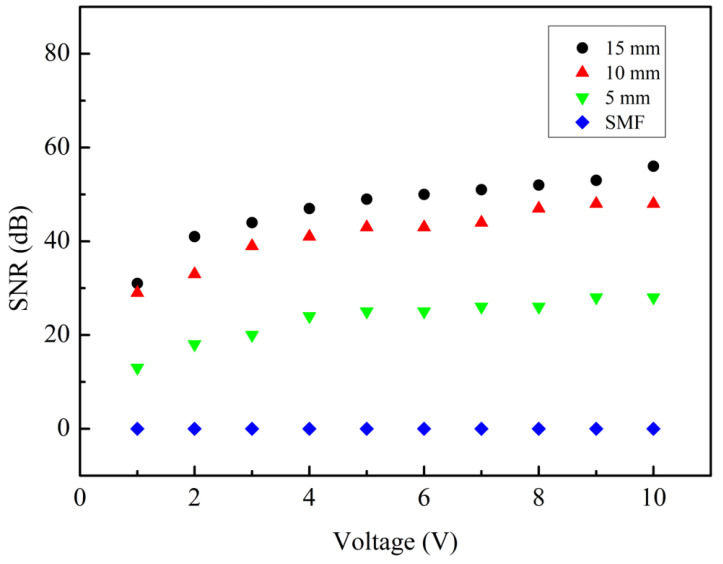
The SNR of sensors with tail fiber lengths of 15, 10, and 5 mm, when the voltage is adjusted from 1 V to 10 V (the frequency is 1000 Hz).

**Figure 8:**
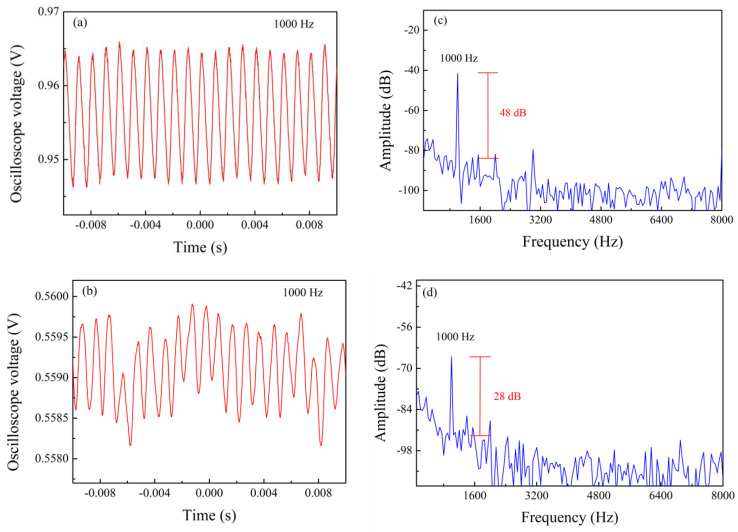
Time domain (**a**,**b**) and frequency domain (**c**,**d**) of sensors with tail lengths of 10 mm and 5 mm when the frequency is 1000 Hz and the voltage amplitude is 10 V.

We also studied the effect of different tail lengths on the sensor’s performance. Based on the above experiments, we selected a sensor with a cavity depth of 75 µm for the test, but the tail length is varied. Vibration sensing experiments were carried out on sensors with tail lengths of 15, 10, and 5 mm successively. The sine wave frequency of the function generator was fixed at 1000 Hz, the voltage amplitude of the function generator varied from 1 V to 10 V, and the experimental data was recorded every 1 V. After FFT was performed on the experimental results, the SNRs of sensors with tail lengths of 15, 10, and 5 mm were obtained, as shown in Figure 7. Figure 8 shows the time domain and frequency domain diagrams of sensors with tail lengths of 10 mm and 5 mm when the function generator voltage amplitude is 10 V. Their SNRs are 48 and 28 dB, respectively. Similarly, we have carried out the above experiments on the unprocessed single-mode fiber, and the obtained single-mode fiber has a signal-to-noise ratio of 0. It can be seen from the figure that the longer the tail length, the higher the SNR of the sensor. At the same time, when the tail length is longer, the mass of the fiber tail acting as the mass block is also larger, which also leads to increased torque. In both cases, the sensor is more prone to deformation, which drives the change of FP cavity length, so the SNR of the sensor becomes larger. Based on this, a sensor with a tail length of 15 mm was selected to continue the following vibration tests.

**Figure 9:**
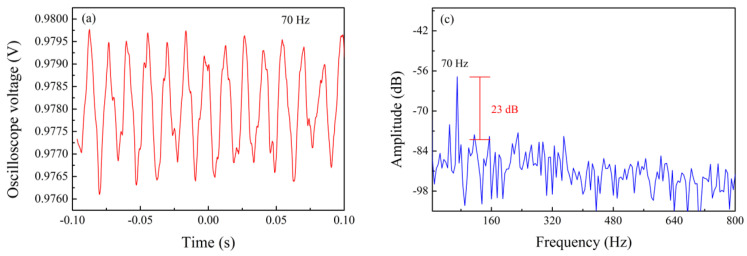

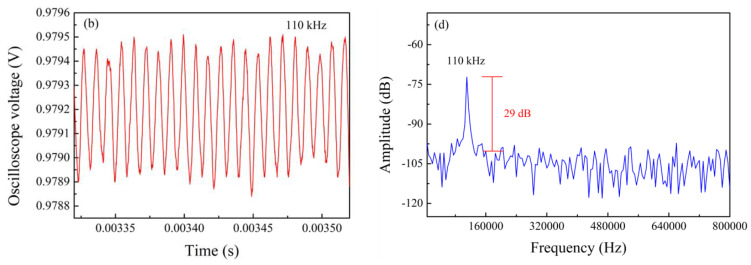
Vibration time domain diagram (**a**,**b**) at 70 Hz and 110 kHz frequencies and frequency domain diagram (**c**,**d**) after FFT.

The frequency measurement range of the sensor was tested with a sensor structure with cavity depth of 75 µm and tail length of 15 mm. A function generator was used to generate a continuous sine wave signal, and the time domain signal of vibration was recorded by an oscilloscope. During the experiment, the amplitude of the function generator was set to 10 V and kept constant, and the function generator was adjusted to generate sinusoidal signals of different frequencies. The experiment found that the sensor could measure the vibration frequency range of 70 Hz–110 kHz. Figure 9 shows the time domain diagram obtained by the oscilloscope when the frequency is 70 Hz and 110 kHz, and the frequency domain diagram after FFT. It can be seen that the obtained frequency signal is consistent with the input frequency signal. Although there is noise, it is easy to separate the main frequency signal from the noise signal. This shows that the sensor can reproduce the vibration signal after loading very well.

**Figure 10:**
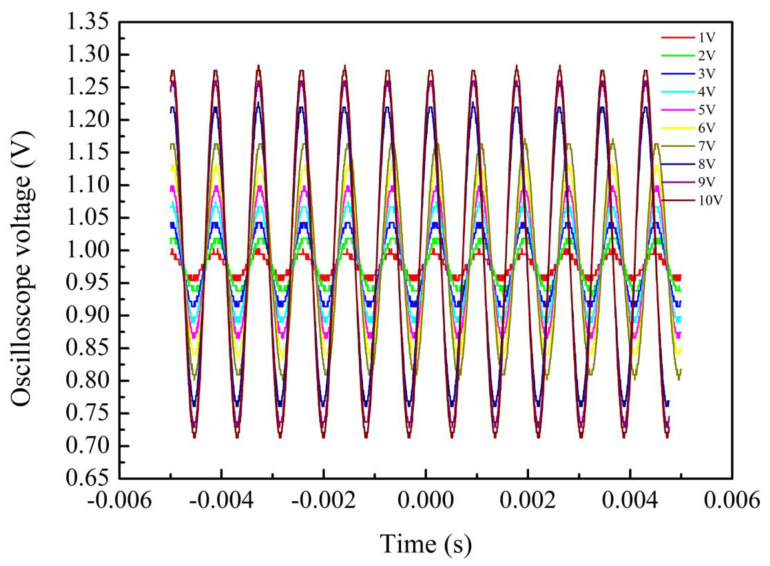
Time domain response of a sensor with a cavity depth of 75 µm and a tail length of 15 mm at different amplitudes.

We also studied the response of the sensor to the amplitude of vibration. The frequency of the function generator was fixed at 1200 Hz, and the amplitude of the input was tuned by changing the voltage amplitude of the function generator. The range of voltage regulation was 1–10 V, with data recorded at 1 V intervals. Figure 10 shows the time-domain curves of output signals of sensors with a cavity depth of 75 µm and a tail length of 15 mm at different amplitudes. The output voltage of the vibration sensor increases with the increase of the driving voltage. This shows that the sensor can recognize vibration signals of different vibration intensities. A phase shift is also observed in the figure, which is due to a phase delay in the electrical module in the vibration sensing system.

**Figure 11:**
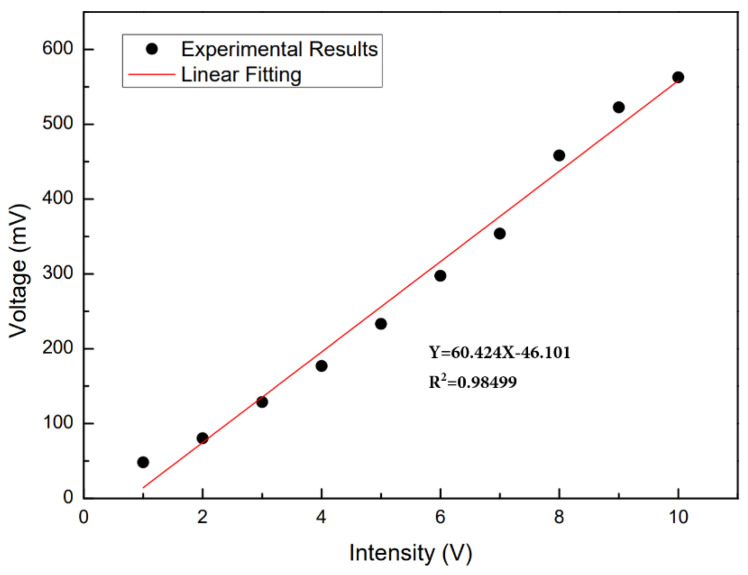
Sensitivity characteristic curve of vibration signal as it increases from 1 V to 10 V.

The peak-to-peak value measured under each driving voltage is taken as the output result of the sensor, and the sensitivity characteristic curve of the vibration sensor to the vibration amplitude is shown in Figure 11. Where the horizontal coordinate is the amplitude voltage of the function generator, and the ordinate is the voltage signal output by the vibration sensor. The relationship between the two is linear, and the sensitivity of the sensor is 60 mV/V.

The fiber FP-cantilever consists of a fiber which has a built-in Fabry-Perot interferometer at its tip. The tip of the fiber can oscillate under the influence of external forces, affecting the length of the interferometer cavity, which in turn modulates the interference pattern of the interferometer.

**Figure 12:**
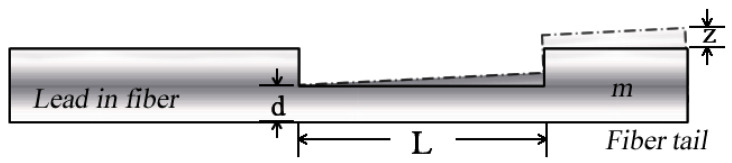
Vibration model of the fiber cantilever beam.

The oscillations of the FP cantilever can be described with a vibration model based on a rectangular cantilever. The movement of the cantilever can be described using the following equation:

(4) $$m\frac{d^{2}z}{dt^{2}}+c\frac{dz}{dt}+kz=F\left(t\right),$$

where m is the mass of the cantilever, c is the damping coefficient, k is the spring constant, z is the deflection of the cantilever beam, and F is the external force acting on the cantilever. Assuming a small vertical deflection, the deflection, z, at the end of the cantilever can be calculated to be [26],

(5) $$z=\frac{L^{3}F}{3ER}$$

where E is Young’s modulus and R is the ratio of the moment of inertia to the mass, which is given by [26],

(6) $$R=\frac{1}{12}wd^{3}\;$$

where w and d denote the width and the thickness of the beam. The change in the FP cavity length, ΔL, can be calculated based on the model in Figure 12, as follows:

(7) $$∆L=L\left(1-\sqrt{1-\frac{z^{2}}{L^{2}}}\right)\;\;\;\;\;=L\left(1-\sqrt{1-\frac{{16L^{4}F}^{2}}{E^{2}w^{2}d^{6}}}\right)$$

**Figure 13:**
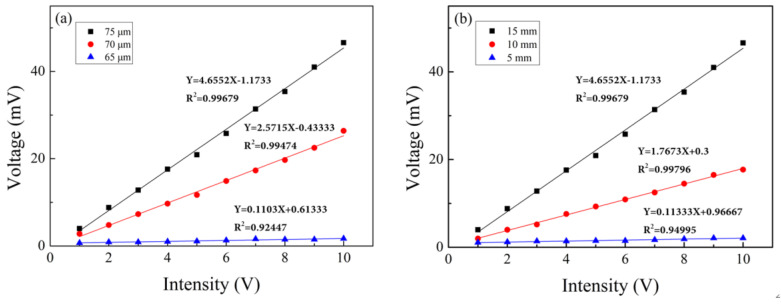
Sensitivity characteristic curves of sensors with different structural parameters. (**a**) Sensitivity characteristic curves of sensors with cavity depths of 75, 70, and 65 µm. (**b**) Sensitivity characteristic curves of sensors with tail lengths of 15, 10, and 5 mm.

The change in cavity length introduces phase shift in the interference Δϕ = 4πΔL/λ, leading to wavelength shift of the spectrum. According to Equation (4), the mass of the fiber tail affects the vibration of the cantilever beam. From Equation (6), one can also tell that the thickness of the cantilever beam has direct influence on the ratio of the moment of inertia to the mass. In order to study the effect of different structural parameters on sensor amplitude sensitivity, sensors with different cavity depths and tail lengths were tested. First, we tested the amplitude responses of sensors with cavity depths of 75, 70, and 65 µm, respectively, while fixing their tail-length at 15 mm. The frequency of the function generator was fixed at 1000Hz, the regulation voltage range from 1V to 10 V, and the data was recorded every 1 V. Figure 13a shows the sensitivity characteristic curve of sensors with cavity depths of 75, 70, and 65 µm. It can be seen that the amplitude sensitivity of the sensor increases with the depth of the cavity. The sensitivity of a sensor with a cavity depth of 75 µm is 1.8 times that of a sensor with a cavity depth of 70 µm, indicating that the cavity depth has a significant impact on the sensitivity of the sensor.

The influence of tail length on sensitivity was also investigated for sensors with tail lengths of 15, 10, and 5 mm, respectively. The frequency of the function generator was fixed at 1000 Hz for testing. The sensitivity curves of sensors with tail lengths of 15, 10, and 5 mm were obtained, as shown in Figure 13b. It can be clearly seen that the longer the tail length, the greater the sensitivity of the sensor. The sensitivity of the sensor with a tail length of 15 mm is 2.6 times that of the sensor with a tail length of 10 mm, indicating that the tail length also has a significant impact on the sensitivity of the sensor.

**Figure 14:**
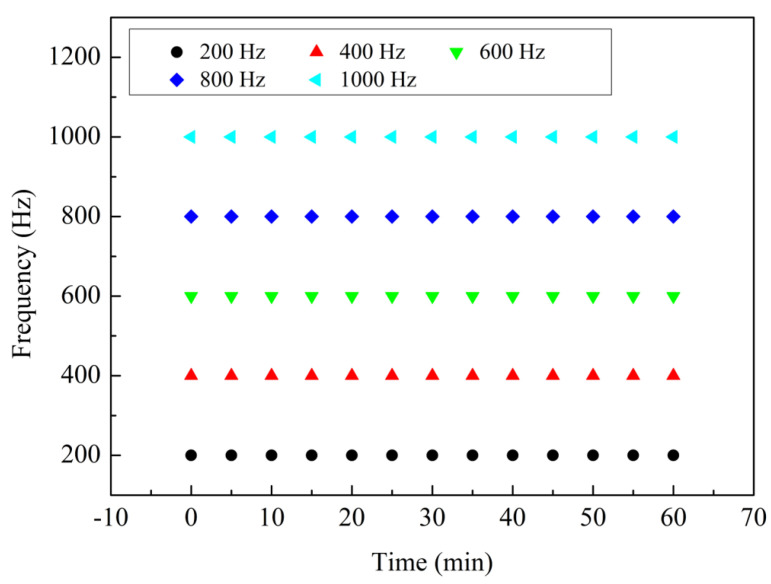
Stability experiments from 0 to 60 min, frequency range 200–1000 Hz.

In addition, the stability of the sensor was investigated experimentally. A sensor with cavity depth of 75 µm and tail length of 15 mm was selected for the experiment. During the experiment, other parameters were kept unchanged, and the sensor was vibrated continuously for 60 min at a fixed frequency. The data on the oscilloscope was recorded every five minutes, and then FFT processing was carried out. The frequency range of the stability experiment was 200–1000 Hz, and the interval of the experimental frequency was 200 Hz. The experimental results are shown in Figure 14, from which one can see that the measured values of each frequency are quite stable.

Table 1 shows a comparison of the proposed sensor with other sensor structures. Compared to other structures, our sensor has a higher amplitude response sensitivity and a wider frequency response range. At the same time, our sensors are less complex and easier to fabricate. In general, our sensor has the unique advantages of wide frequency response range, high sensitivity, low preparation difficulty, compact structure, and low cost, making it a promising choice in the field of vibration sensing in the future.

## 4. Conclusions

In this article, we proposed a novel all-fiber vibration sensor structure based on a fiber Fabry-Perot interferometer (FPI), which is fabricated using femtosecond laser technology. We systematically studied the effects of various structural parameters on the sensor’s performance. The experimental results demonstrate that a sensor with a cavity depth of 75 µm and a tail length of 15 mm exhibits the best performance, achieving the highest signal-to-noise ratio (SNR) and sensitivity. The sensor’s detection frequency range spans from 70 Hz to 110 kHz, with an SNR of 56 dB at 1000 Hz. Additionally, the sensitivity was measured to be 60 mV/V at 1200 Hz. The sensor boasts several advantages, including a compact structure, straightforward manufacturing process, low cost, high sensitivity, and a wide measurement range. These attributes make it a promising candidate for future applications in vibration sensing.

## Figures and Tables

**Table 1:** Comparison of sensing performance with other optical fiber sensing structures.

Year	Structure	Frequency Range	Sensitivity	**Complexity**
2018	BSMZI [19]	10–20 Hz	13.575 dB/w	Medium
2018	Flexible FPI [20]	200 Hz–97 kHz	0.088 mV/mPa	High
2023	FP cavity encapsulated by silica film [21]	200–400 Hz	4.2 mV/V	High
2023	SMF-DCF-SMF [22]	0.1 Hz–47 kHz	Unknown	Medium
2019	Photonic Crystal Fiber [23]	10 Hz–20 kHz	Unknown	High
2017	FPI with a D-shaped silica ferrule [27]	200 Hz–12.5 kHz	0.121 mV/mPa	High
2024	Our work	70 Hz–110 kHz	60 mV/V	Low

## Data Availability

Data are available from the corresponding author and can be provided upon appropriate request.
